# Integrative enrichment analysis of gene expression based on an artificial neuron

**DOI:** 10.1186/s12920-021-00988-x

**Published:** 2021-08-25

**Authors:** Xue Jiang, Weihao Pan, Miao Chen, Weidi Wang, Weichen Song, Guan Ning Lin

**Affiliations:** 1 Shanghai Mental Health Center, Shanghai Jiao Tong University School of Medicine, School of Biomedical Engineering, Shanghai Jiao Tong University, Shanghai, 200030 China; 2 Shanghai Key Laboratory of Psychotic Disorders, Shanghai, 200030 China

**Keywords:** Huntington’s disease, Multi-tissues, Differentially expressed gene, Artificial neuron

## Abstract

**Background:**

Huntington’s disease is a kind of chronic progressive neurodegenerative disease with complex pathogenic mechanisms. To data, the pathogenesis of Huntington’s disease is still not fully understood, and there has been no effective treatment. The rapid development of high-throughput sequencing technologies makes it possible to explore the molecular mechanisms at the transcriptome level. Our previous studies on Huntington’s disease have shown that it is difficult to distinguish disease-associated genes from non-disease genes. Meanwhile, recent progress in bio-medicine shows that the molecular origin of chronic complex diseases may not exist in the diseased tissue, and differentially expressed genes between different tissues may be helpful to reveal the molecular origin of chronic diseases. Therefore, developing integrative analysis computational methods for the multi-tissues gene expression data, exploring the relationship between differentially expressed genes in different tissues and the disease, can greatly accelerate the molecular discovery process.

**Methods:**

For analysis of the intra- and inter- tissues’ differentially expressed genes, we designed an integrative enrichment analysis method based on an artificial neuron (IEAAN). Firstly, we calculated the differential expression scores of genes which are seen as features of the corresponding gene, using fold-change approach with intra- and inter- tissues’ gene expression data. Then, we weighted sum all the differential expression scores through a sigmoid function to get differential expression enrichment score. Finally, we ranked the genes according to the enrichment score. Top ranking genes are supposed to be the potential disease-associated genes.

**Results:**

In this study, we conducted large amounts of experiments to analyze the differentially expressed genes of intra- and inter- tissues. Experimental results showed that genes differentially expressed between different tissues are more likely to be Huntington’s disease-associated genes. Five disease-associated genes were selected out in this study, two of which have been reported to be implicated in Huntington’s disease.

**Conclusions:**

We proposed a novel integrative enrichment analysis method based on artificial neuron (IEAAN), which displays better prediction precision of disease-associated genes in comparison with the state-of-the-art statistical-based methods. Our comprehensive evaluation suggests that genes differentially expressed between striatum and liver tissues of health individuals are more likely to be Huntington’s disease-associated genes.

**Supplementary Information:**

The online version contains supplementary material available at 10.1186/s12920-021-00988-x.

## Background

Huntington’s disease (HD) is a representative neurodegenerative disease, caused by excessive triplet (CAG) repeat located in huntingtin (HTT) gene on chromosome 4 that codes for polyglutamine in the huntingtin protein [1]. The mutant protein has many effects in cells through entering the nucleus followed by gene transcription changes [2]. With accumulation of the mutant protein, numerous interactions between molecules and a number of molecular pathways are affected, resulting in neuronal dysfunction and degeneration [3, 4]. With the connections between neurons getting sparse, the neurons start dying gradually, and finally died during the disease deterioration. At the meanwhile, the volume of striatum decreased markedly [5]. HD can lead to motor, cognitive, and emotional impairments progressively. The molecular pathogenesis of HD is very complicated. It has been reported that many pathogenic factors may be related to the disease, such as neurotrophasthenia, impairment of axon transmission, impairment of metabolic pathways, protein misfolding, inflammation, and intestinal microorganism [6–11]. However, the molecular mechanisms of HD can not be completely explained by a single pathogenic factor. To date, the complicated molecular pathogenesis of HD still remains elusive.

To elucidate the molecular pathological mechanisms, researchers in biomedical research field are focusing on the study of biomarkers and the regulatory pathways related to specific phenotype of chronic diseases. This traditional hypothesis-based researches need long research periods and high labor cost. However, with the rapid and encouraging development of high-throughput sequencing technologies, such as RNA-seq, ATAC-seq, ChIP-seq, and RIP-seq, many computational methods and softwares for investigating the molecular targets have been developed in recent years. Therefore, researchers could firstly screen biomarkers or catch a glimpse of the interactions between molecules from a genome-wide scale using computational methods with omics data, and then use online resources to obtain functional annotations or pathway information. It is obvious that the results obtained in bioinformatics provide a useful reference for biomedical researchers.

The computational methods can be roughly divided into three major categories: network-based methods [12–16], statistics-based enrichment analysis methods [17–19], and machine learning based methods [20–22]. Meanwhile, network-based methods can well describe the regulatory relationship between regulatory elements and interaction sites in the DNA sequence from the system level. Network-based methods often need large amounts of computational time. Statistics-based enrichment analysis methods compute the statistical difference p values using case samples and control samples. Machine learning based methods, such as matrix factorization based methods [23, 24] and deep learning methods [25, 26], have been widely used and studied in the field of bio-medicine recently. Matrix factorization based methods may lead to unstable results due to random initialization, and deep learning methods often need large amounts of samples to train the predictive models.

The complex molecular pathological mechanisms and complicated phenotypes of neurodegenerative diseases provide great challenges for screening disease-associated genes. On the one hand, there is still a large gap between the interpretation of pathological mechanisms and the disease-associated genes screened by various computational methods. On the other hand, the consistency of the candidate disease-associated gene sets obtained by different methods is poor [25]. What’s more, we found that it is very difficult to distinguish disease-associated genes from non-disease genes of Huntington’s disease in our previous study [23]. At present, it is urgent to develop effective computational methods to improve the accuracy of disease-associated gene prediction and the robustness of the candidate disease-associated gene sets, promoting the understanding of the pathological molecular mechanisms under complex phenotypes.

Genes often selectively expressed in different tissues. Recent studies in the field of bio-medicine have shown that the molecular origin of chronic complex diseases may not exist in the diseased tissue. Differentially expressed genes between different tissues are expected to reveal the molecular origin of complex chronic diseases [27–30]. Previously, researchers usually screen genes that significantly differentially expressed between normal and case samples of different individuals as disease-associated ones. However, because of the fact that large amounts of genes’ expression have been affected during the disease development, it becomes quite difficult to accurately distinguish disease-associated genes from non-disease essential genes. Besides, due to the gene selectively expressed in different tissues, different gene sets can be obtained by using samples of different tissues. Moreover, the differentially expressed genes selected with normal and case samples may not helpful for the personalized medicine due to the individual differences. Nevertheless, exploring the differentially expressed genes between different tissues of a same individual may reveal endogenous answers for the disease development.

According to the above analysis, in this study, we conducted large amounts of experiments to screen Huntington’s disease-associated genes using classical methods, including t-test [31], fold change method (FC) [31], flexible non-negative matrix factorization method (FNMF) [23], and joint non-negative matrix factorization meta-analysis method (jNMFMA) [24], to explore the relationship of differentially expressed genes and the disease.

To further improve the disease-associated gene prediction accuracy, we conducted a meta-analysis of the differential expression scores of a gene, including intra-tissues’ differential expression scores (i.e. differential expression score between different tissues) and inter-tissues’ differential expression scores (i.e. differential expression score between normal samples and case samples of one tissue). Hence, we designed an integrative enrichment analysis of intra- and inter- tissues’ differentially expression scores of one gene based on an artificial neuron (IEAAN).

Firstly, we calculated the differential expression scores of a gene using FC [31] approach. The differential expression scores are seen as features of the gene. Then, we integrated the differential expression scores to get an enrichment score of the corresponding gene using an artificial neuron model. Finally, we prioritized disease-associated genes according to the enrichment score. Experiments on gene expression data of Huntington’s disease show that the prediction accuracy of IEAAN could be as precise as that of the-state-of-art methods. Furthermore, the gene rankings in ranked list of IEAAN are more stable than that of other methods. More importantly, IEAAN is much helpful for understanding the mechanisms under complex disease phenotypes and have provided insight into the molecular mechanisms underlying Huntington’s disease.

The rest of this paper is organized as follows: The IEAAN approach proposed in this study is presented in “Methods” section. Experiments that screen differentially expressed genes with RNA-seq data of Huntington’s disease are illustrated and the overall discussion of experimental results of various methods is reported in “Results and discussion” section. Conclusions are presented in “Conclusions” section.

## Methods

In this section, we present the key idea of the integrative enrichment analysis, and then describe the details of the artificial neuron model and the learning process. Finally, the parameter setting of the IEAAN is discussed.

### Integrative enrichment analysis

Enrichment analysis aims to select a set of genes which are significantly differentially expressed between different conditions. The resulting gene set is considered to be strongly correlated with the accuracy of distinguishing one condition from the others. Traditional enrichment analysis methods were used to evaluate the significance of gene set using statistical-based strategy. Then the corresponding gene set was assigned an enrichment score which was used to measure the importance of the gene set.

Intuitively, the interpretability and biological meaning could be further improved if we integrate all the differential expression scores of one gene. Machine learning methods and deep learning methods are suitable for data integration and prediction. So, according to the above analysis, we designed the integrative enrichment analysis model based on an artificial neuron to intergrate the differential expression scores of one gene.

### Integrative enrichment analysis of intra- and inter- tissues’ differentially expressed genes based on an artificial neuron

#### Model

The gene intra-tissues’ differential expression scores and inter-tissues’ differential expression scores were computed based on FC [31]. It should be ensured that the fold change of any two samples must be not less than 1. If not, the reciprocal is used. The differential expression score (greater score indicates that the gene is more significantly differentially expressed) of a gene is the average fold changes of any two samples from normal ones and case ones respectively.

Symbol $x_g = (p_{g1},\cdots ,p_{gn})$ represents the differential expression scores of gene *g*. $p_{gi}$ represents the differential expression score obtained by the $i-th$ method. In this study, the differential expression scores $p_{gi}, i=1,\cdots ,n$ were seen as features of gene *g*. We trained the artificial neuron with the genes in the training set. The labels of genes are denoted as $Y=(y_1,\cdots ,y_g)$. The value of $y_g$ is defined by

1 $$\begin{aligned} y_g = {\left\{ \begin{array}{ll} 1, &{} \mathrm{if~g~is~disease-associated},\\ -1, &{} \mathrm{if~g~is~non-disease-associated}. \end{array}\right. } \end{aligned}$$

**Fig. 1:**
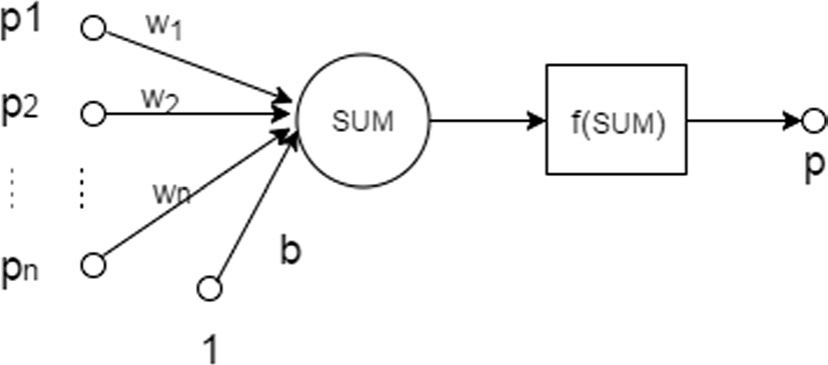
Schematic illustration of the integrative enrichment analysis artificial neuron model

**Fig. 2:**
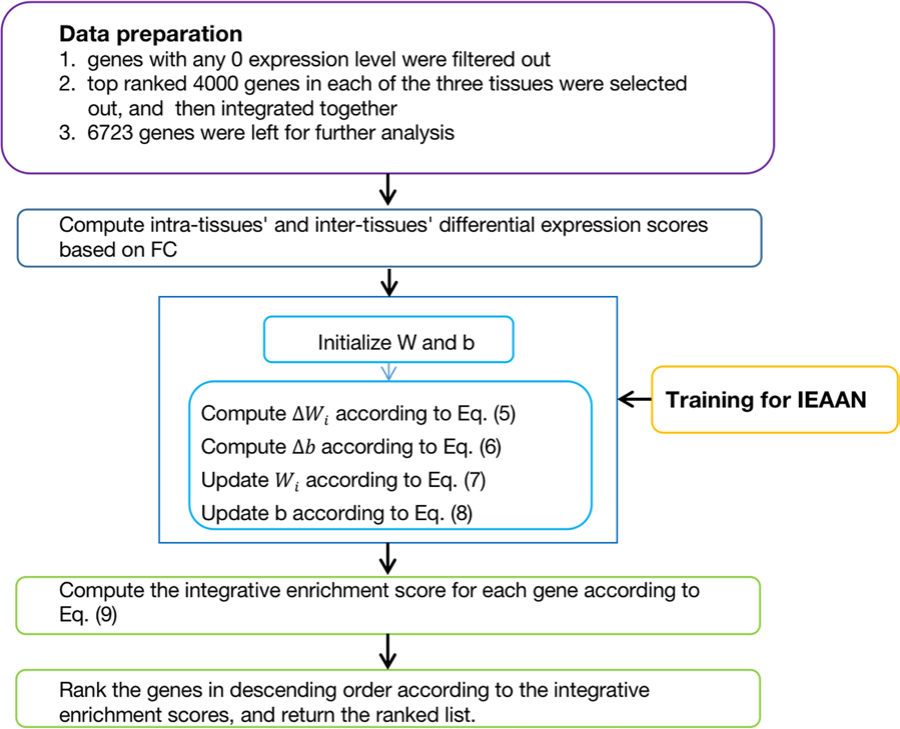
Pipeline of the integrative analysis of gene expression data based on the IEAAN model. Data preparation and pre-processing steps are described firstly. The training process of the IEAAN are illustrated in detail. Disease-associated genes are prioritized according to the enrichment scores finally

**Fig. 3:**
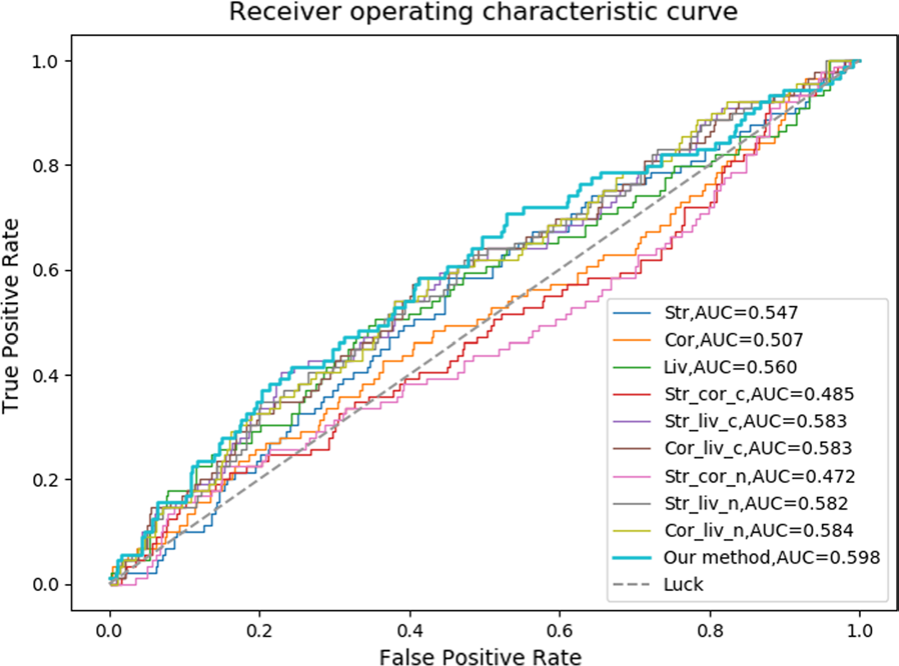
The ROC curves of FC-based results

**Fig. 4:**
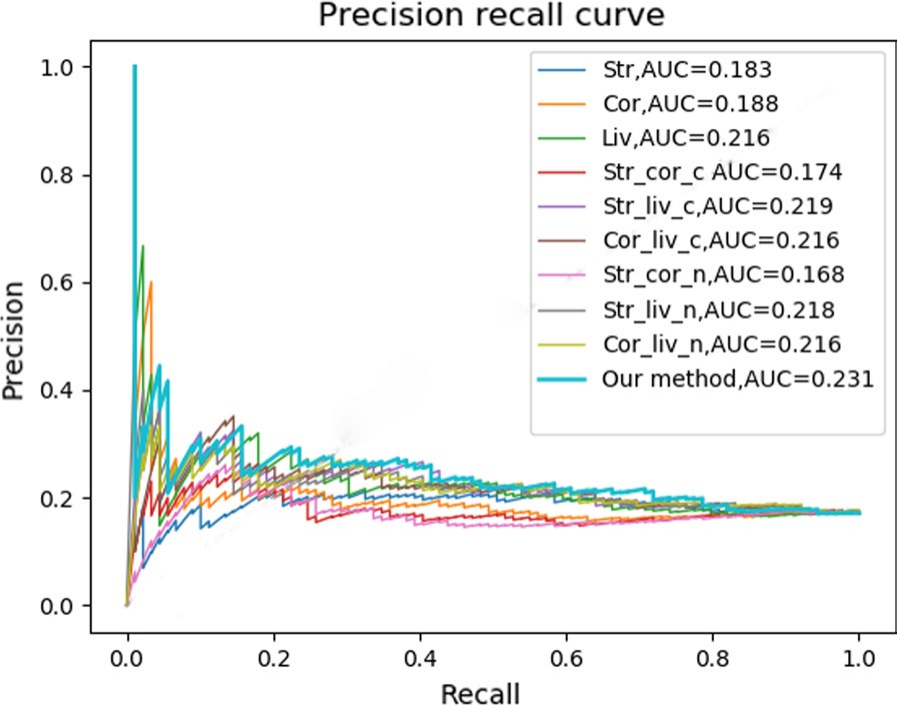
The PR curves of FC-based results

In the artificial neuron model (Fig. 1), $p_i$ represents the differential expression score, and $w_i$ and *b* are the parameters of the model. Sigmoid function is used as activation function to integrate all the features of a gene. The sigmoid function is written as

2 $$\begin{aligned} f_{\theta }(x_g)= \frac{1}{1+e^{-(\sum _{i=1}^{n}w_ip_{gi}+b)}}, \end{aligned}$$

where $\theta =(W,b)$ represents the parameter setting.

Let $\hat{y_g} = f_{\theta }(x_g)$ represents the evaluated label of gene *g*. Mean square error is used as loss function, which is written as

3 $$\begin{aligned} L(Y,{\hat{Y}})= \sum _{g=1}^{N}l(y_g,\hat{y_g}), \end{aligned}$$

where *N* represents the number of genes.

Therefore, the loss function for gene *g* is defined by

4 $$\begin{aligned} l(y_g,\hat{y_g})= \frac{1}{2}(y_g-\hat{y_g})^2. \end{aligned}$$

#### Learning

In this study, error back-propagation algorithm is used to learn parameters in the model. The learning process is completed until the loss function converges. The gradient descent algorithm is used to calculate the gradient in each iteration. The calculation of gradient is given by

5 $$\begin{aligned} \Delta w_i&= \frac{\partial L}{\partial w_i} \nonumber \\&= \sum _{g=1}^{N}\frac{\partial l_g}{\partial w_i} \nonumber \\&= -\sum _{g=1}^{N}(y_g-\hat{y_g})\frac{e^{-(\sum _{i=1}^{n}w_ip_{gi}+b)}}{(1+e^{-(\sum _{i=1}^{n}w_ip_{gi}+b)})^2}{p_{gi}}, \end{aligned}$$

6 $$\begin{aligned} \Delta b r= \frac{\partial L}{\partial b} \nonumber \\&= \sum _{g=1}^{N}\frac{\partial l_g}{\partial b} \nonumber \\&= -\sum _{g=1}^{N}(y_g-\hat{y_g})\frac{e^{-(\sum _{i=1}^{n}w_ip_{gi}+b)}}{(1+e^{-(\sum _{i=1}^{n}w_ip_{gi}+b)})^2}. \end{aligned}$$

7 $$\begin{aligned} W(k+1)&= W(k) - \eta \Delta W(k), \end{aligned}$$

8 $$\begin{aligned} b(k+1)&= b(k) - \eta \Delta b(k). \end{aligned}$$

where *W*(*k*) is the weighted matrix of the model in the *k*-th interactions. $\eta$ is the learning rate, which controls the convergent speed.

### Integrative enrichment score

Finally, Eq. (9) was used to calculate integrative enrichment score for each gene. We ranked the genes in descending order according to the integrative enrichment score. Top ranking genes are more likely to be disease-associated genes.

9 $$\begin{aligned} E_g = \sum _{i=1}^{n}w_ip_{gi} . \end{aligned}$$

By training the artificial neuron, we can clearly know which differential expression score contributes more to the finally integrative enrichment score according to the weight, providing a better understanding of the relationship between disease phenotype and the differentially expressed genes.

### Parameter setting

Here, we initialized parameters in the model as follows: 1) if the area under the receiver operating characteristic curve (AUC) [32] of the differential expression score is larger than 0.5, the corresponding *w* is preset to be the AUC, 2) the other *w* is preset to be 0, 3) the *b* is preset to be 0.

The integrative enrichment analysis model described here is generally applicable to any high-dimensional sequencing data for meaningful biological biomarkers discovery.

## Results and discussion

In Fig. 2, we present the pipeline of steps needed to develop for high-precision biomarker discovery from the whole-genome gene expression data to downstream analysis.

Next, we first described the dataset used in this study in detail. Second, we demonstrated the experimental results of four common used methods. Then, we analyzed and discussed the disease-associated gene prediction accuracy of four traditional methods and the IEAAN. The robustness of IEAAN was also further tested by randomly selecting samples. Finally, 5 genes were selected out by integrating the results of IEAAN and those of FC-based experiments.

### Gene expression data

Gene expression data used in this study were downloaded at http://www.hdinhd.org, which were obtained from 6-month-old Huntington’s disease mice through RNA-seq technology. The dataset contains three tissues: striatum, cortex, and liver. There are 6 kinds of genotypes, including ploy Q20, ploy Q80, ploy Q92, ploy Q111, ploy Q140, and ploy Q175. For each genotype, there are 8 samples. The genotype ploy Q20 is the normal one, while the other genotypes are disease ones. Altogether there are 144 samples and there are 23351 genes for each sample in the dataset. The detailed information of the dataset is illustrated in Table 1.

**Table 1:** Experimental data description

Age	6-month-old					
Tissue	Striatum	Cortex	Liver			
Genotype	poly Q20	poly Q80	poly Q92	poly Q111	poly Q140	poly Q175
Total sample number	144					

Since most of the computational methods select disease genes from transcript level based on the hypothesis that disease-associated genes tend to be significantly differently expressed in case samples compared with normal ones. Genes whose expression changed slightly during the disease development are difficult to be selected out. Therefore, we conducted a filter step to reduce computational complexity. First, genes with any 0 expression level were filtered out according to *l*0-norm. Then, gene expression through samples was normalized, and the genes were ranked in descending order according to the variance in striatum, cortex, and liver respectively (Additional file 1 Figure S1, Additional file 2 Figure S2, and Additional file 3 Figure S3, respectively). The top ranked 4000 genes have larger expression variances, compared with the relatively small expression variances of the other genes. Due to the fact that computational methods have no discriminative ability for the genes with small variance, the top ranked 4000 genes in each of the three tissues were manually selected out and then integrated together. Finally, 6,723 genes were selected out from the whole genome for next analysis.

The modifier genes were from [33, 34]. There were 520 genes, including 89 disease genes and 431 non-disease genes.

### Prediction performance of t-test, FC, FNMF, and jNMFMA

It has been reported that large amounts of genes’ expression, as well as the interactions between genes, are affected during the Huntington’s disease progression. The pathological molecular mechanisms of HD are still unclear. In this study, we conducted experiments using t-test, FC, FNMF, and jNMFMA, to explore the characteristics of intra-tissues’ differentially expressed genes and inter-tissues’ differentially expressed genes, respectively. We denoted the experiments using normal samples (gene expression data with genotype Q20) versus normal samples as Normal-Normal, the experiments using normal samples versus case samples (gene expression data with genotype Q80, Q92, Q111, Q140, or Q175) as Normal-Case, and the experiments using case samples versus case samples as Case-Case.

For the non-parameter methods, i.e., t-test and FC, we conducted the experiment once to obtain the stable gene ranking results. Due to the instability of methods with many parameters that need to be randomly initialized, i.e., FNMF and jNMFMA, we conducted experiments 10 times. Then, the mean and standard deviation of the 10 experimental results were calculated as the final assessment. The experimental results for the four methods are shown in Tables 2, 3, 4, and 5, respectively.

**Table 2:** Performance of the t-test method

t-test	Normal–case			Normal–normal			Case–case		
	Str_Str	Cor_Cor	Liv_Liv	Str_Cor	Str_Liv	Cor_Liv	Str_Cor	Str_Liv	Cor_Liv
AUC	0.48	0.52	0.47	0.545	0.529	0.512	0.521	0.514	0.523
AUPR	0.16	0.17	0.15	0.200	0.186	0.178	0.178	0.178	0.183

**Table 3:** Performance of the FC method

FC	Normal–case			Normal–normal			Case–case		
	Str_Str	Cor_Cor	Liv_Liv	Str_Cor	Str_Liv	Cor_Liv	Str_Cor	Str_Liv	Cor_Liv
AUC	0.55	0.51	0.56	0.472	0.582	0.584	0.485	0.583	0.583
AUPR	0.18	0.19	0.23	0.168	0.218	0.216	0.174	0.219	0.216

**Table 4:** Performance of the jNMFMA method

jNMFMA	Normal–case			Normal–normal			Case–case		
	Str_Str	Cor_Cor	Liv_Liv	Str_Cor	Str_Liv	Cor_Liv	Str_Cor	Str_Liv	Cor_Liv
AUC	0.567	0.554	0.585	0.527	0.534	0.548	0.537	0.581	0.563
	±0.016	±0.005	±0.021	±0.013	±0.011	±0.029	±0.023	±0.009	±0.014
AUPR	0.207	0.194	0.216	0.181	0.191	0.196	0.187	0.221	0.206
	±0.015	±0.005	±0.011	±0.006	±0.008	±0.012	±0.019	±0.009	±0.009

**Table 5:** Performance of the FNMF method

FNMF	Normal–case			Normal–normal			Case–case		
	Str_Str	Cor_Cor	Liv_Liv	Str_Cor	Str_Liv	Cor_Liv	Str_Cor	Str_Liv	Cor_Liv
AUC	0.554	0.556	0.569	0.542	0.566	0.537	0.540	0.549	0.545
	±0.016	±0.014	±0.029	±0.015	±0.022	±0.029	±0.016	±0.032	±0.032
AUPR	0.199	0.197	0.194	0.194	0.192	0.198	0.188	0.195	0.191
	±0.017	±0.010	±0.016	±0.008	±0.013	±0.024	±0.009	±0.013	±0.011

From Tables 2 and 3, we can know that the t-test and FC perform poorly in disease-associated gene prediction with intra-tissues’ Normal-Case samples, while they perform better with both inter-tissues’ Normal-Normal samples and inter-tissues’ Case-Case samples. It indicates that the differentially expressed genes of inter-tissues are more likely to be disease-associated genes. It also indicates that it is easily to screen disease-associated genes with Normal-Normal samples or Case-Case samples. Besides, the performance of the two methods with inter-tissues’ Normal-Normal samples is comparable to that with inter-tissues’ Case-Case samples. It demonstrates that the differentially expressed genes between different tissues in health individuals are likely to be disease-associated genes.

Comparing Tables 2 and 3, we observed that the FC method outperformed t-test method. We reasoned that this difference might be because t-test method uses the average information of gene expression, ignoring lots of useful information, and finally leads to poor result.

Tables 4 and 5 show the experimental results using jNMFMA method and FNMF method. The two methods have similar performance in screening disease-associated genes under various conditions.

Through comprehensive comparison of Tables 2, 3, 4, and 5, we found that the performance of the jNMFMA and FNMF methods were superior than that of the two statistical-based methods when screening differentially expressed genes with intra-tissues’ gene expression data. However, there was no statistical significance among the results of the 4 methods with inter-tissues’ gene expression data. These results indicate that genes differentially expressed among inter-tissues in healthy individuals have great relationship with the disease, providing a new perspective on disease-associated gene screening.

### Performance comparison of IEAAN with other four methods

We further analyzed the performance of IEAAN and the other four methods. For jNMFMA and FNMF, we conducted 10 time experiments with random initialization. The best performed experiment was used to conduct comparison analysis. We observed that jNMFMA and FNMF performed better at prediction accuracy and prediction precision for top ranking genes, with Normal-Case samples of striatum, cortex, and liver, respectively (Additional file 4 Figure S4 and Additional file 5 Figure S5). Moreover, FC, jNMFMA, and FNMF have similar performances, which are better than t-test method (Additional file 6 Figure S6, Additional file 7 Figure S7, Additional file 8 Figure S8, and Additional file 9 Figure S9). It is because that t-test solely uses the average expression of gene, possibly missing some useful information. However, due to the random initialization, jNMFMA and FNMF produce unstable final ranked lists of genes. Moreover, jNMFMA and FNMF display higher computational complexity.

From above results, we concluded that FC, as a parameterless method, is relatively simple, effective, and stable. With the gene expression data considered in details, FC may yet better results. Therefore, we designed IEAAN to integrate differential expression scores obtained by FC method. The results of IEAAN and FC are shown in Figs. 3, and 4.

Compared with the best result of FC, the AUC was improved by 2.6% in IEAAN (AUC=0.598), and in the meanwhile, the AUPR was improved by 5.4% IEAAN (AUPR=0.231). To test the robustness of the method, we randomly took out 2 samples, then computed the intra-tissues’ and inter-tissues’ differential expression scores based on FC with the left 6 samples. The IEAAN model was re-run with those differential expression scores as the features of gene. The procedure has been repeated for 5 times. The final ranking lists of the 5 experiments have been analyzed and the overlap degree of the top ranked 500 genes was 0.73, suggesting the robustness and stability of the integrative model.

Moreover, to verify the consistency of the gene ranked lists, which are obtained from the differential expression scores using FC with intra-tissues’ gene expression data and inter-tissues’ gene expression data, we analysed the overlap degree of top ranking genes between any two ranked lists. Since the prediction precision of disease-associated genes is very high when the recall rate is no more than 0.10, we checked the rankings of top 9 ($89 * 0.10 = 8.90$) genes in the ranked lists, and found that they are ranked in top 800 of the final ranked lists. So, we statistics the overlap degree of the top 800 genes, and found that the overlap degrees were larger than 0.20 Table 6. The overlap degree between the result of IEAAN and that of FC with better performance is higher, while the one between the result of IEAAN and that of FC with poor performance was lower. The above analysis results indicate that genes differentially expressed between tissues are more susceptible to be affected and differentially expressed during the disease progression.

**Table 6:** The overlap degree of the top 800 genes in any two ranked lists obtained by FC

		Normal–case			Normal–normal			Case–case		
		Str_Str	Cor_Cor	Liv_Liv	Str_Cor	Str_Liv	Cor_Liv	Str_Cor	Str_Liv	Cor_Liv
Normal–case	Cor_Cor	0.38								
	Liv_Liv	0.22	0.19							
Normal–normal	Str_Cor	0.48	0.26	0.21						
	Str_Liv	0.25	0.14	0.44	0.27					
	Cor_Liv	0.21	0.13	0.45	0.21	0.92				
Case–case	Str_Cor	0.39	0.29	0.41	0.87	0.26	0.20			
	Str_Liv	0.25	0.14	0.44	0.27	0.96	0.91	0.26		
	Cor_Liv	0.21	0.12	0.45	0.20	0.91	0.96	0.19	0.92	
IEAAN	–	0.23	0.14	0.46	0.23	0.93	0.95	0.23	0.93	0.95

Integrating the top 800 gene sets of the eight ranked lists, we finally obtained 5 genes simultaneously presented in the top 800 of all ranked lists. They were Arpp21 (cAMP-regulated phosphoprotein 21), Rgs4 (regulator of G-protein signaling 4), Rasd2 (RASD family member 2), Gabrd (gamma-aminobutyric acid type A receptor delta subunit), and Tmod1 (tropomodulin 1). These five genes were found to be differentially expressed in intra-tissues and inter-tissues. The functional annotations of the five genes are shown in Table 7. Among the five differential expressed genes, Arpp21 is related to cellular response to heat and nucleic acid binding, and Rgs4 is involved in inactivation of MAPK activity and GTPase activator activity. Rasd2 has been previously implicated in synaptic transmission and GTP binding, Gabrd is related to cell junction and GABA-A receptor complex, and Tmod1 can play a role in muscle contraction and pointed-end action filament capping (Table 7). By investigating the prefrontal cortex single cell expression, it was found that Arpp21, Rasd2, Gabrd, Tmod1 mainly express in astrocytes, neurons, microglia, OPC, stem cells and GABAergic neurons, while Rgs4 mainly expresses in neurons, OPC, stem cells, and GABAergic neurons.

The five genes may play an key role during HD progression. It is important to note that Arrpp21 and Rasd2 have also been reported in the article [33], suggesting the effectiveness of IEAAN and the significance of the five genes for the disease development.

**Table 7:** The functional annotations of the five genes

Gene	GOTERM_BP_DIRECT	GOTERM_CC_DIRECT	GOTERM_MF_DIRECT
Arpp21	Cellular response to heat	Cytoplasm	Nucleic acid binding
Rgs4	Inactivation of MAPK activity	Nucleus	GTPase activator activity
	Regulation of G-protein coupled	Cytoplasm	
	Receptor protein signaling pathway		
Rasd2	Synaptic transmission	Intracellular	GTP binding
	Dopaminergic	Membrane	
	Small GTPase mediated signal transduction		
Gabrd	Transport	Plasma membrane	GABA-A receptor activity
	Ion transport	Membrane	Extracellular ligand-gated ion channel activity
	Signal transduction	Integral component of membrane	
		Cell junction	
		Synapse	
		GABA-A receptor complex	
Tmod1	Muscle contraction	COP9 signalosome	Tropomyosin binding
	Adult locomotory behavior	Membrance	
	Myofibril assembly	sarcomere	
	Pointed-end action filament capping	Cortical cytoskeleton	
	Lens fiber cell development		

The results indicate that the-state-of-art methods could not effectively distinguish the disease genes from non-disease ones. To improve the performance of disease gene selection tools, in this study, we developed a integrative computational methods from a new perspective to mine the differentially expressed genes between different tissues of healthy individuals. Finally, we obtained five disease-related genes by prioritizing the differentially expressed genes between different tissues. The best performance of AUC is around 0.6, and AUPR is around 0.23. It suggests that our method can also be very helpful for understanding the endogenous reasons of disease.

## Conclusions

Prioritizing differentially expressed genes as disease-associated genes can not perform well in the Huntington’s disease gene expression data analysis. To better understand molecular mechanisms under complicated phenotypes, we designed IEAAN to integrate the differential expression scores of intra-tissues’ and inter-tissues’. In this study, we conducted extensive experiments to analyze the performance of different methods with different samples. We demonstrated that differentially expressed genes between different tissues of healthy individuals are likely to be disease-associated genes. We finally screened five genes, including Arpp21, Rgs4, Rasd2, Gabrd, and Tmod1, two (Arpp21 and Rasd2) of which have been reported to be related with Huntington’s disease [33].

## Supplementary Information

**Additional file 1**. Genes ranked in descending order according to the gene expression variance in Striatum tissue.

**Additional file 2**. Genes ranked in descending order according to the gene expression variance in Cortex tissue.

**Additional file 3**. Genes ranked in descending order according to the gene expression variance in Liver tissue.

**Additional file 4**. The receiver operating characteristic curve of t-test, FC, FNMF, and jNMFMA with Normal-Case samples of striatum, cortex, and liver, respectively.

**Additional file 5**. The precision recall curve of t-test, FC, FNMF, and jNMFMA with Normal-Case samples of striatum, cortex, and liver, respectively.

**Additional file 6**. The receiver operating characteristic curve of t-test, FC, FNMF, and jNMFMA with Normal-Normal samples of striatum, cortex, and liver, respectively.

**Additional file 7**. The precision recall curve of t-test, FC, FNMF, and jNMFMA with Normal-Normal samples of striatum, cortex, and liver, respectively.

**Additional file 8**. The receiver operating characteristic curve of t-test, FC, FNMF, and jNMFMA with Case-Case samples of striatum, cortex, and liver, respectively.

**Additional file 9**. The precision recall curve of t-test, FC, FNMF, and jNMFMA with Case-Case samples of striatum, cortex, and liver, respectively.

## Data Availability

The gene expression data used in this study were downloaded from http://www.hdinhd.org. To make the dataset available to public, we deposit it in publicly available repository, please download at https://figshare.com/s/171c8ade2e7051556356, https://figshare.com/s/c74ac543e4893e283259, and https://figshare.com/s/ae4575a6185f6326e710. The modifier genes were from “Langfelder P, Cantle J P, Chatzopoulou D, et al. Integrated genomics and proteomics define huntingtin CAG length-dependent networks in mice. Nature Neuroscience, 2016. PMID: 26900923 DOI: 10.1038/nn.4256”. We also deposit it in publicly available repository, please download at https://figshare.com/s/13fdc5c17d736142dcd0. It is important to noted that two (Arpp21 and Rasd2) of our findings in this study have also been reported in “Langfelder P, Cantle J P, Chatzopoulou D, et al. Integrated genomics and proteomics define huntingtin CAG length-dependent networks in mice. Nature Neuroscience, 2016. PMID: 26900923 DOI: 10.1038/nn.4256”.

## Abbreviations

FC: Fold change
FNMF: Flexible non-negative matrix factorization
jNMFMA: Joint non-negative matrix factorization meta-analysis method
ROC: Receiver operating characteristic
PR: Precision-recall
AUC: The area under the ROC curve
AUPR: The area under the PR curve
