# Supplementary figures and images for: Integrative enrichment analysis of gene expression based on an artificial neuron
Source: BMC Med Genomics. 2021 Aug 25;14(Suppl 1):173. doi: 10.1186/s12920-021-00988-x (PMC8386081; doi:10.1186/s12920-021-00988-x)

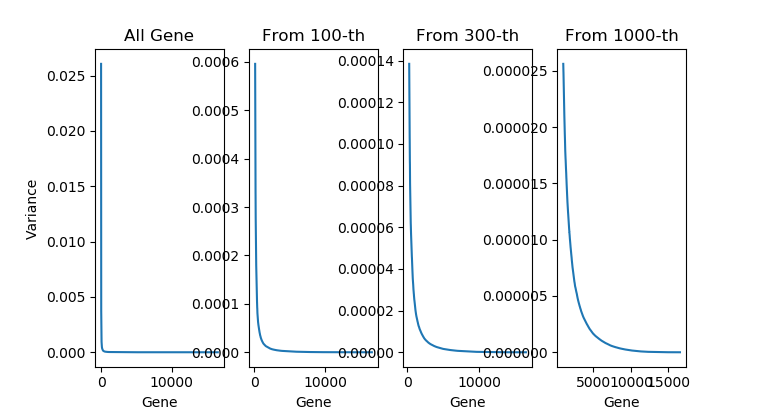

Supplement: Supplementary file 1 — Additional file 1. Genes ranked in descending order according to the gene expression variance in Striatum tissue. [file 12920_2021_988_MOESM1_ESM.png]

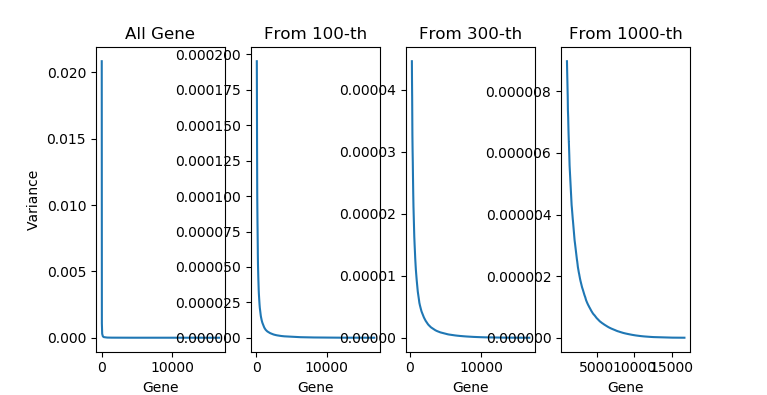

Supplement: Supplementary file 2 — Additional file 2. Genes ranked in descending order according to the gene expression variance in Cortex tissue. [file 12920_2021_988_MOESM2_ESM.png]

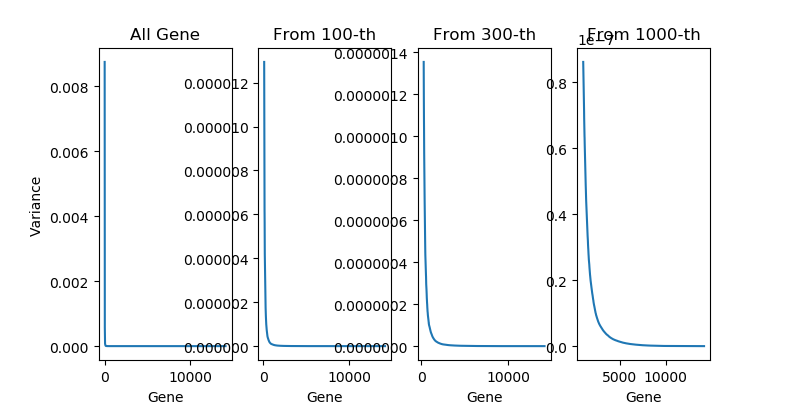

Supplement: Supplementary file 3 — Additional file 3. Genes ranked in descending order according to the gene expression variance in Liver tissue. [file 12920_2021_988_MOESM3_ESM.png]

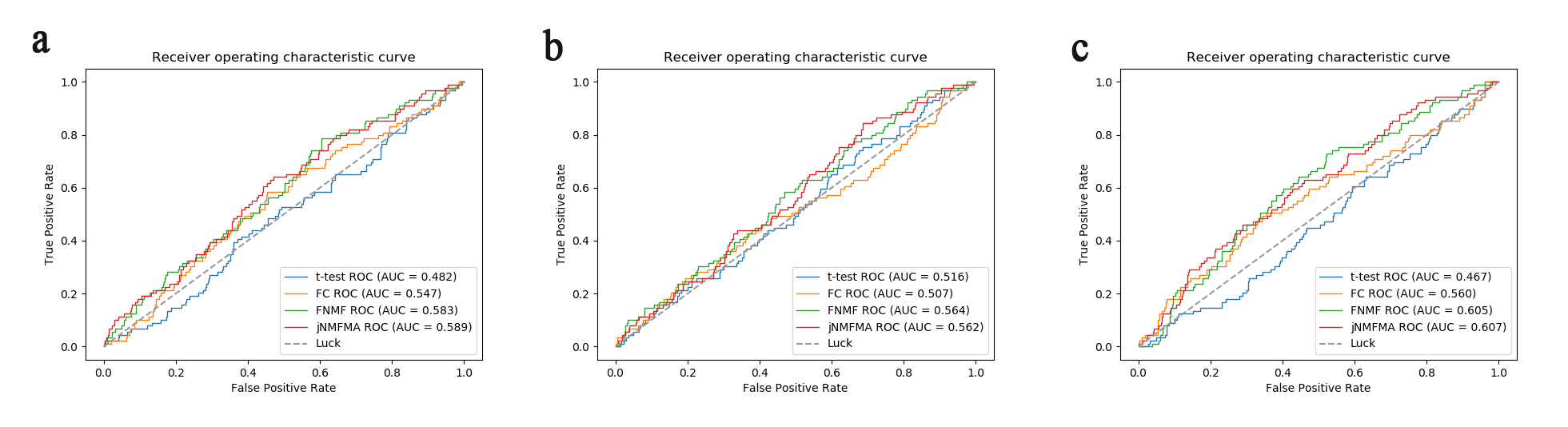

Supplement: Supplementary file 4 — Additional file 4. The receiver operating characteristic curve of t-test, FC, FNMF, and jNMFMA with Normal-Case samples of striatum, cortex, and liver, respectively. [file 12920_2021_988_MOESM4_ESM.png]

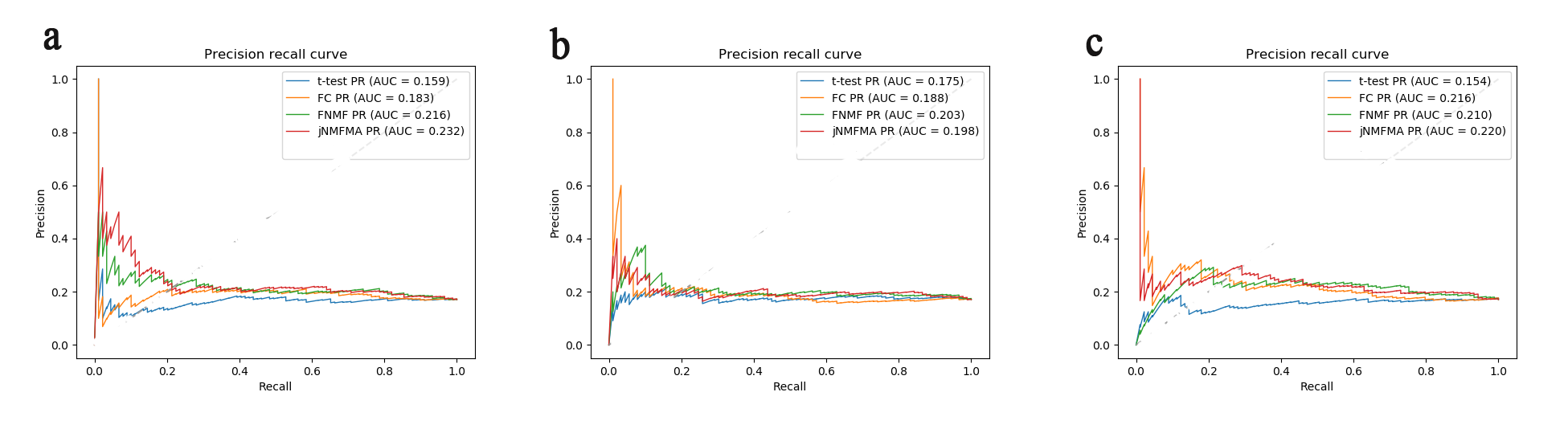

Supplement: Supplementary file 5 — Additional file 5. The precision recall curve of t-test, FC, FNMF, and jNMFMA with Normal-Case samples of striatum, cortex, and liver, respectively. [file 12920_2021_988_MOESM5_ESM.png]

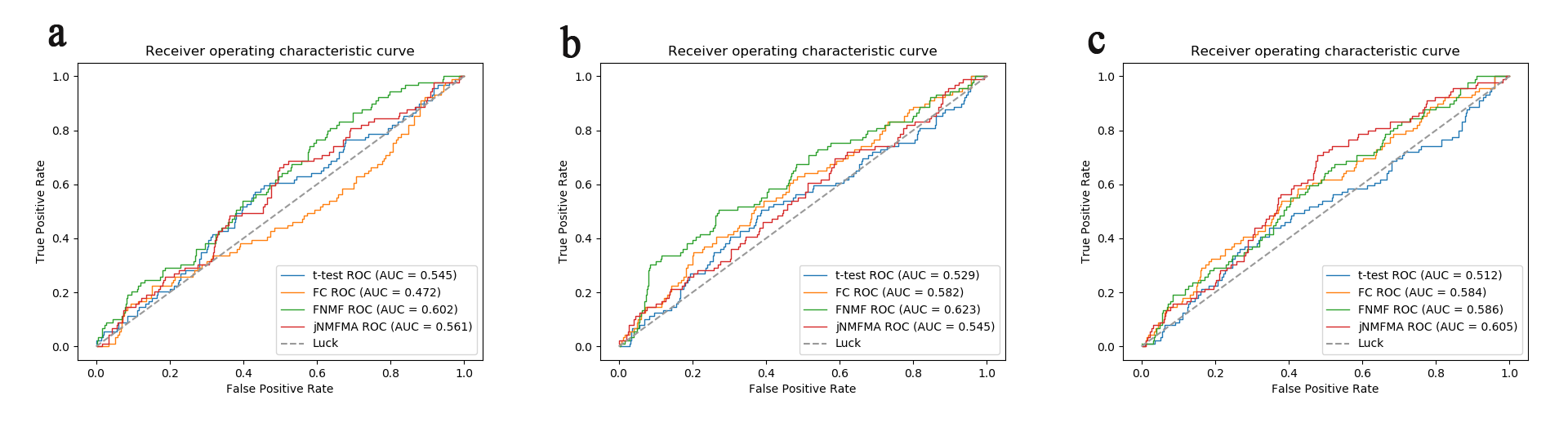

Supplement: Supplementary file 6 — Additional file 6. The receiver operating characteristic curve of t-test, FC, FNMF, and jNMFMA with Normal-Normal samples of striatum, cortex, and liver, respectively. [file 12920_2021_988_MOESM6_ESM.png]

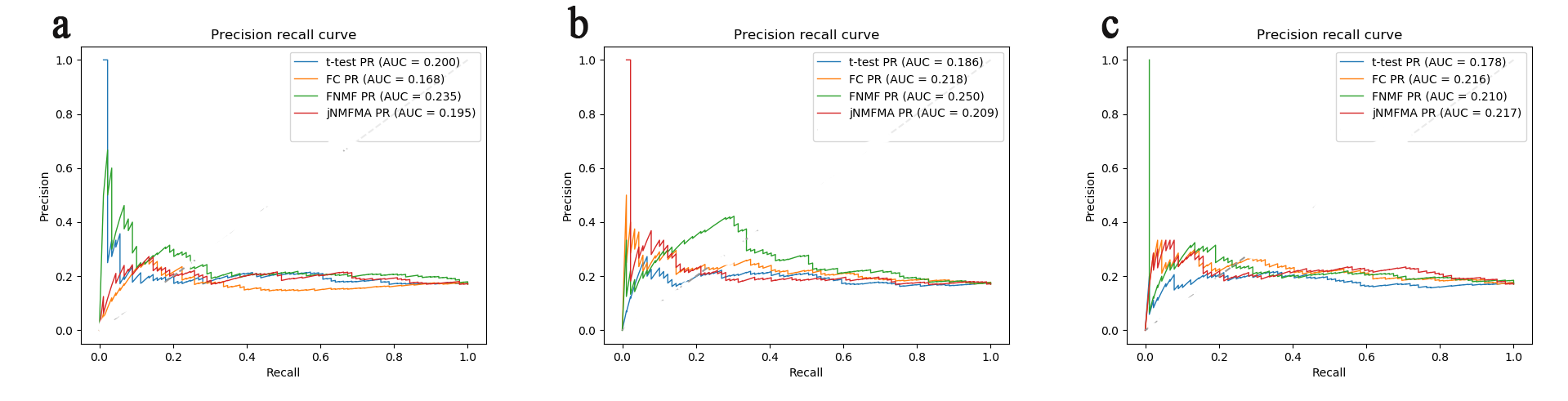

Supplement: Supplementary file 7 — Additional file 7. The precision recall curve of t-test, FC, FNMF, and jNMFMA with Normal-Normal samples of striatum, cortex, and liver, respectively. [file 12920_2021_988_MOESM7_ESM.png]

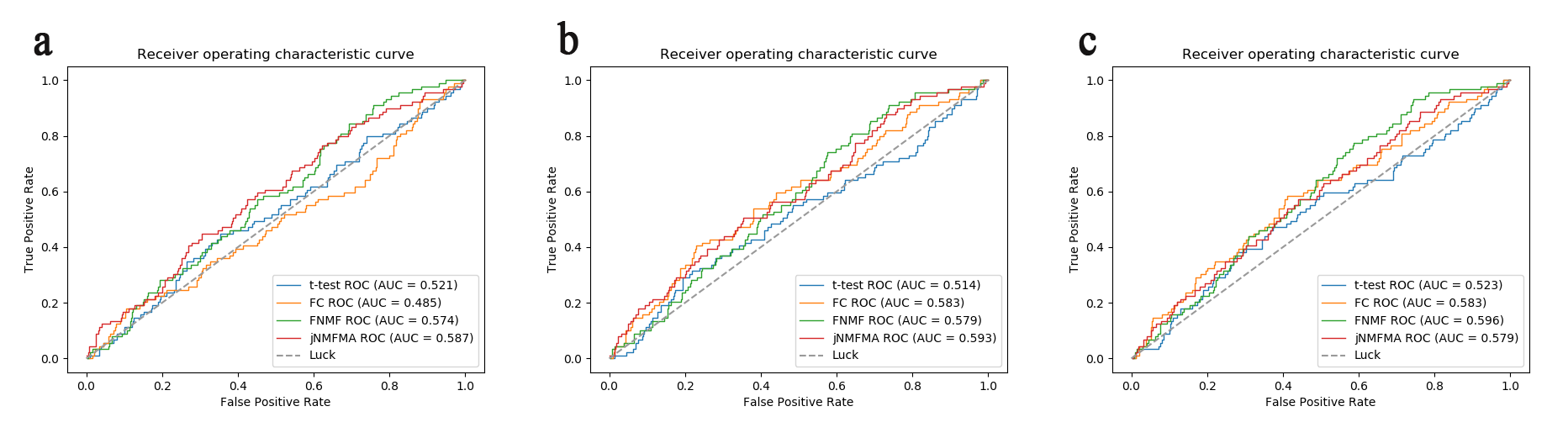

Supplement: Supplementary file 8 — Additional file 8. The receiver operating characteristic curve of t-test, FC, FNMF, and jNMFMA with Case-Case samples of striatum, cortex, and liver, respectively. [file 12920_2021_988_MOESM8_ESM.png]

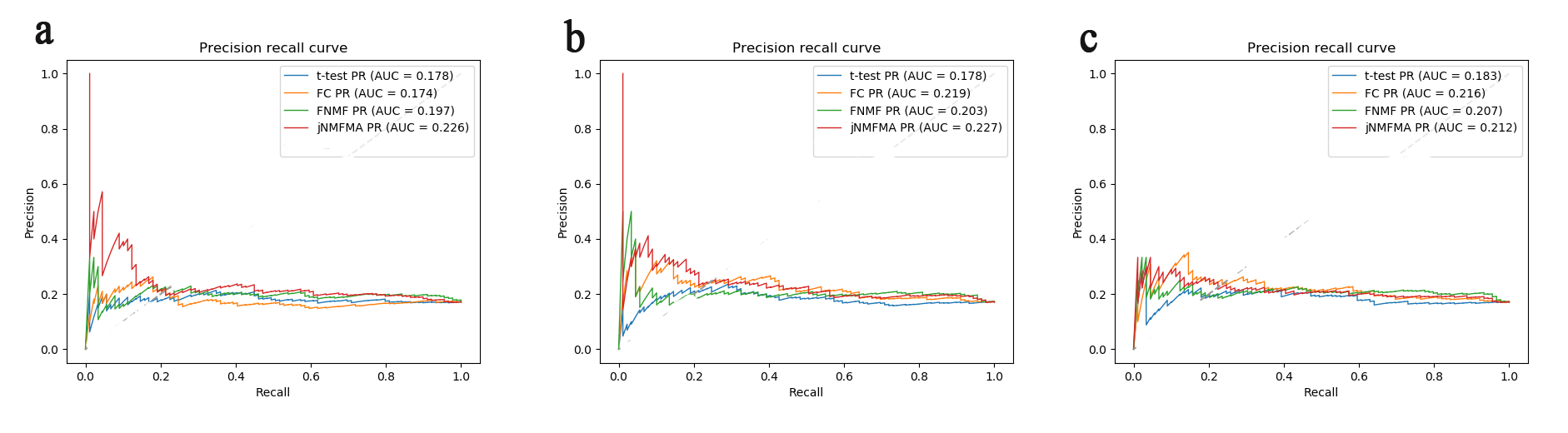

Supplement: Supplementary file 9 — Additional file 9. The precision recall curve of t-test, FC, FNMF, and jNMFMA with Case-Case samples of striatum, cortex, and liver, respectively. [file 12920_2021_988_MOESM9_ESM.png]
